# Modification of the surface nanotopography of implant devices: A translational perspective

**DOI:** 10.1016/j.mtbio.2021.100152

**Published:** 2021-10-21

**Authors:** K. Harawaza, B. Cousins, P. Roach, A. Fernandez

**Affiliations:** Chemistry Department, School of Science, Loughborough University, Loughborough, LE11 3TU, UK

**Keywords:** Nanotopography, Clinical translation, Clinical trials, Surface modification, Implants

## Abstract

There is an increasing need for the development of superior, safe, and more sophisticated implants, especially as our society historically has been moving towards an increasingly aging population. Currently, most research is being focused on the next generation of advanced medical implants, that are not only biocompatible but have modified surfaces that direct specific immunomodulation at cellular level. While there is a plethora of information on cell-surface interaction and how surfaces can be nanofabricated at research level, less is known about how the academic knowledge has been translated into clinical trials and commercial technologies. In this review, we provide a clinical translational perspective on the use of controlled physical surface modification of medical implants, presenting an analysis of data acquired from clinical trials and commercial products. We also evaluate the *state-of-the-art* of nanofabrication techniques that are being applied for implant surface modification at a clinical level. Finally, we identify some current challenges in the field, including the need of more advanced nanopatterning techniques, the comparatively small number of clinical trials and comment on future avenues to be explored for a successful clinical translation.

## Graphical abstract

**Figure:**
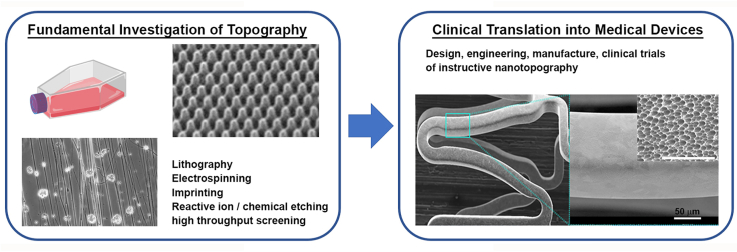

## 1 Introduction

The demand for implantable devices is rising dramatically due to several persistent factors, such as the explosive growth of an ageing population accompanied with an increasing number of chronic conditions. As an example, the worldwide demand for medical implants is expected to account for more than $613B in 2025, with an annual growth on average of 5.4% during this decade [1]. Notably, the evolution of these devices has contributed to extend our life expectancy and improve our wellbeing. However, implant failure still represents a big challenge, requiring in most cases either revision after 10–15 years or removal surgery. Hip implant failure, for example, represents only 1% of the total hip implants [2] but in other fields such as dental, this figure increases dramatically up to 10% in some procedures [3]. Foreign body response (FBR) and bacterial infection are amongst the main causes of implant failure [4]. Nevertheless, each failed implant represents a huge economic burden on health care services and societal impact, which translates in an increasing need for the development of safer and more sophisticated implantable devices. New regulatory environments such as the newly introduced EU Medical Device Regulation 2017/745 (MDR) are expected to include fundamental changes that are aimed to increase patient safety and health outcomes at an expense of introducing more regulatory burdens for future developments on medical devices [5,6].

In the last decade, research efforts have largely focussed on biomaterials that provide medical implants with increased adaptability and complexity that can stimulate a very specific cellular response at the molecular level; this class is largely understood as a 4th generation of biomaterials [7]. Such a specific molecular response translates into a more sophisticated immunomodulation in response to the implant surface-tissue interaction [8,9] A more precise control of the events leading to protein adsorption and immunomodulation [10,11] can be achieved by a careful design of the physicochemical surface properties of implantable biomaterials such as topography [12], wettability [13], chemistry (charge, energy) [14,15], light [16], mechanical and visco-elastic properties [17,18]. Surface topographical modification is an adaptable strategy since does not require a change in the bulk material nor a chemical alteration of the surface [19], which reduces the probabilities of chemical toxicity [20]. Topographical modification of biomaterials is an attractive research field with unique cell-surface interactions characteristic of the parameters presented by surface chemistry, topography, biological conditioning and cell mediation of the local environment. The level of interest and developments within this rapidly expanding field are evidenced by the number of experimental articles and reviews published in recent years [21,22]. Multiple studies have shown that different nanostructures induce a diverse range of synergistic interactions between cells and the surface of the biomaterial, including mechanical actuation and electrophysiological activity of certain cell types, e.g., nanokicking [23].

Due to advances in technology and biophysical understanding, there are a number of current topical reviews outlining chemical and physical modification of biomaterial surfaces from a fundamental perspective [[24], [25], [26]]. Most of these reviews are focused on the fabrication techniques employed at a developmental level (largely within academia) and particularly focused on understanding the micro- and nano-scale characteristics impacting upon biological response [20,27]. However, despite of the vast amount of fundamental work, the clinical translation of these developments remains slow, with a lack of visibility on how fundamental knowledge in this area moves into clinical application. There is an urgent need for a focussed review in this field, providing a basis to help navigate change. Here, we will introduce a translational perspective on the surface modification of implantable devices, including exemplary studies that have reached the milestone of clinical trials or commercial stage. This review is divided into several sections: the first section is dedicated to describe the process of immunomodulation and the state-of-the-art technologies employed to fabricate the surface topographies of different biomaterials. We also include insight on the high-throughput screening approaches for the purpose of optimising the design and dimensions of nano-topographical surface features and their *in vitro* response. The second section is dedicated to summarize case examples of topographically designed surfaces that have reached the clinical or commercial stage, and the nanofabrication techniques used. Finally, we give a concise appraisal of the current translational aspects and discuss possible future perspectives on the horizon.

## 2 The acid test of medical device surface-tissue interaction: a controlled immunomodulation

**Fig. 1:**
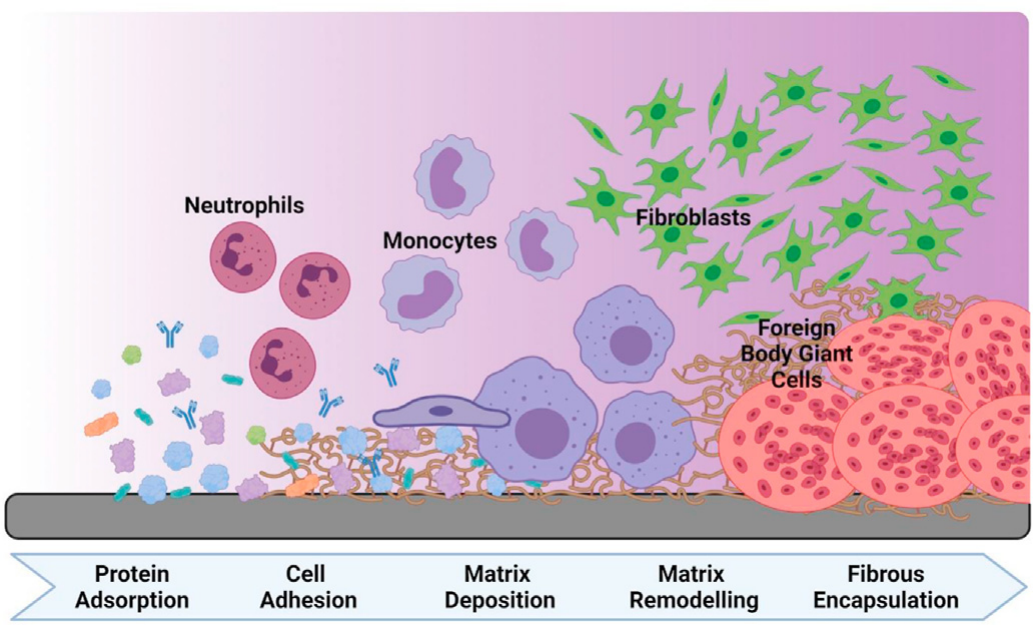
Response to implant. Temporal representation of the events following medical device implantation, including a selection of the cell types and the complexity of the interactions/matrix composition occurring over time (left to right).

Foreign body response (FBR) to implanted devices is the major reason why many biomedical implants fail, in most cases requiring patients to undergo revision surgery or removal [28,29]. After a biomaterial is implanted into the body (Fig. 1), various non-specific proteins adsorb to the surface of the material simultaneously [30,31], which triggers a cascade of inflammatory and wound-healing responses [32]. Neutrophils are the first to infiltrate the area and become activated as they adsorb to the protein monolayer and release cytokines, chemokines, reactive oxygen species and various other enzymes [33]. Next, the cascade invites tissue-residing monocytes which differentiate into macrophages. These macrophages proceed to release their own chemical signals and attract fibroblast cells, which in turn produce excessive amounts of collagen. The resulting extracellular matrix-rich layer encapsulates the biomaterial and forms a substrate layer with which monocytes and macrophages can interact [34]. By fusing together to form foreign body giant cells a continuous fibrous layer can be formed leading to encapsulation of the medical device. Cell-material interactions, combined with the surrounding interstitial fluid, can lead to implant failure (e.g., corrosion, leaching and breakdown products lead to inflammation/remodelling, thrombosis, etc.) [10,35]. Regulation of type 1 and type 2 macrophages is necessary to modulate anti- and pro-inflammatory responses, impacting on the remodelling of tissue at the implant interface [36]. Finally, a fibrotic capsule is formed around the material, causing implant failure and the subsequent need for repair surgery.

The body of research focussed on surface chemical and/or topography effects on tissue interaction, either alone or working in synergy, is vast, with many articles highlighting the critical natura of fine-tuning surface parameters to control the dynamic tissue-implant interface [37,38]. The current review focusses on the clinical translation of these findings, as such it is useful to point out some of the recent advances for context. Osteoimmunomodulation, for instance, is a widely investigated interface with nanotopography effects being shown to control the osteoimmune environment. Changes in the expression of inflammatory cytokines, osteoclastic activities, and osteogenic, angiogenic, and fibrogenic factors have been observed in relation to surface chemical changes (amine or acrylic acid functionaliation) acting in synergy with nanoparticle surfaces (in the diameter range 16, 38, and 68 ​nm) [39]. Similarly, *in vitro* surface studies have shown nanotopography effects on primary neutrophil and macrophage responses, leading to enhanced matrix metalloproteinase-9 production from primary neutrophils, and a decrease in pro-inflammatory cytokine secretion from primary macrophages [40]. These changes are likely resulting from the pre-adsorbed protein layer forming at the substrate interface, with a good breadth of understanding now focussed on this area also. At the molecular-scale proteins can unfold and deform to stabilise interaction with material surfaces, thus presenting different biochemical environments for subsequent cell-mediation [31,41,42].

It is clear that the responses occurring at the material surface underpin the dynamic changes occurring during interaction with the biological milieu. Pre-clinical assessment of these materials and their surfaces is therefore critical for early evaluation. There are a plethora of *in vitro* assessments, being targeted largely for broad-spread viability assessment for cell interaction or tissue integration. Many of these studies will look at initial interactions, with those intended for medical devices also requiring more stringent approaches to assess longer-term effects [43], e.g. metal leaching [44]. To give brief context to this, many materials/surface modifications emerge from fundamental research specifically focussed for use within a particular tissue type, e.g., hemocompatible vs biocompatible; bone compared to neural tissue. The definitions selected for compatibility must always take into account the intended use and longevity of effects observed. [7,45] These early studies, more often than not, highlight key factors of specific interest for that tissue, addressing for instance if neurons attach and elongate along surface topographical features [46,47]. High throughput approaches have been reviewed for *in vitro* assessment, giving a cost-time benefit for materials screening, but again have limitations in terms of their biological assessment focus [48]. All of the pre-clinical assessment have their advantages and limitations, and must be selected to enable safe adoption of the material/medical device into clinical application.

The factors that affect the degree at which FBR occurs is dependent on the physicochemical surface characteristics presented by the implanted material, such as porosity, shape, size, and roughness [49,50]. Therefore, it is possible to control the FBR by altering the surface nanotopography that could closely mimic the *in vivo* physiological environment [51]. Moreover, surface parameters such as height and width, also affect the cell response and can have drastic effects on cell phenotype [52]. Thus, it is evident that cells are extremely sensitive to changes in nanotopographical features; however, the precise mechanism as to why this is the case, remains unclear [53].

## 3 Nanofabrication techniques

Since its conception, the biomedical field has benefitted from cross-disciplinary activity and sharing of both knowledge and instrumentation towards nanofabrication. These techniques were largely developed for application in other industries, such as semiconductors and optics [54,55]. As it stands, there are multiple techniques used in academia and industry for the fabrication of nano-surfaces; a broad selection of commonly used techniques is presented in Table 1, with various advantages and limitations depending on the intended use and scale of operation*.* From these techniques, nanostructures with a diverse range of sizes and geometries can be fabricated, including designed surface roughness. Some important parameters to consider when deciding to select a nanofabrication technique needed for a commercial application, are the degree of pattern control and resolution, cost of ownership, scale-up potential, and biomaterials versatility. There are several criteria to follow for the classification of nanofabrication techniques. Here, we consider process parameters to categorise nanofabrication techniques between methods that can produce regular patterns and methods for surface roughening.

**Table 1:** Summary of various nanofabrication method used in academia and industry to obtain regular patterns and surface roughening for biological applications.

Nanofabrication method	Cost of Ownership	Scale-up potential	Degree of Pattern Control	Material versatility	Main user (Academia/Industry)
Photolithography	Very Expensive	Unlimited	Unlimited	Very limited	Academia
Electron beam lithography	Expensive	Very limited	Unlimited	Very limited	Academia
Nanoimprinting	Expensive	Unlimited	Unlimited	Very limited	Both
Chemical etching	Affordable	Unlimited	Very limited	Limited	Both
Reactive ion etching	Moderate	Unlimited	Very limited	Limited	Industry
Electrospinning	Moderate	Limited	Very limited	Wide range	Both
Gas-cluster ion beam	Moderate	Unlimited	Very limited	Wide range	Industry
Selective laser sintering	Very expensive	Very limited	Very limited	Limited	Industry
Plasma spray	Affordable	Unlimited	Very limited	Wide range	Industry
Sandblasting	Affordable	Unlimited	Very limited	Wide range	Industry

### 3.1 Topographic regular nanopatterning

**Fig. 2:**
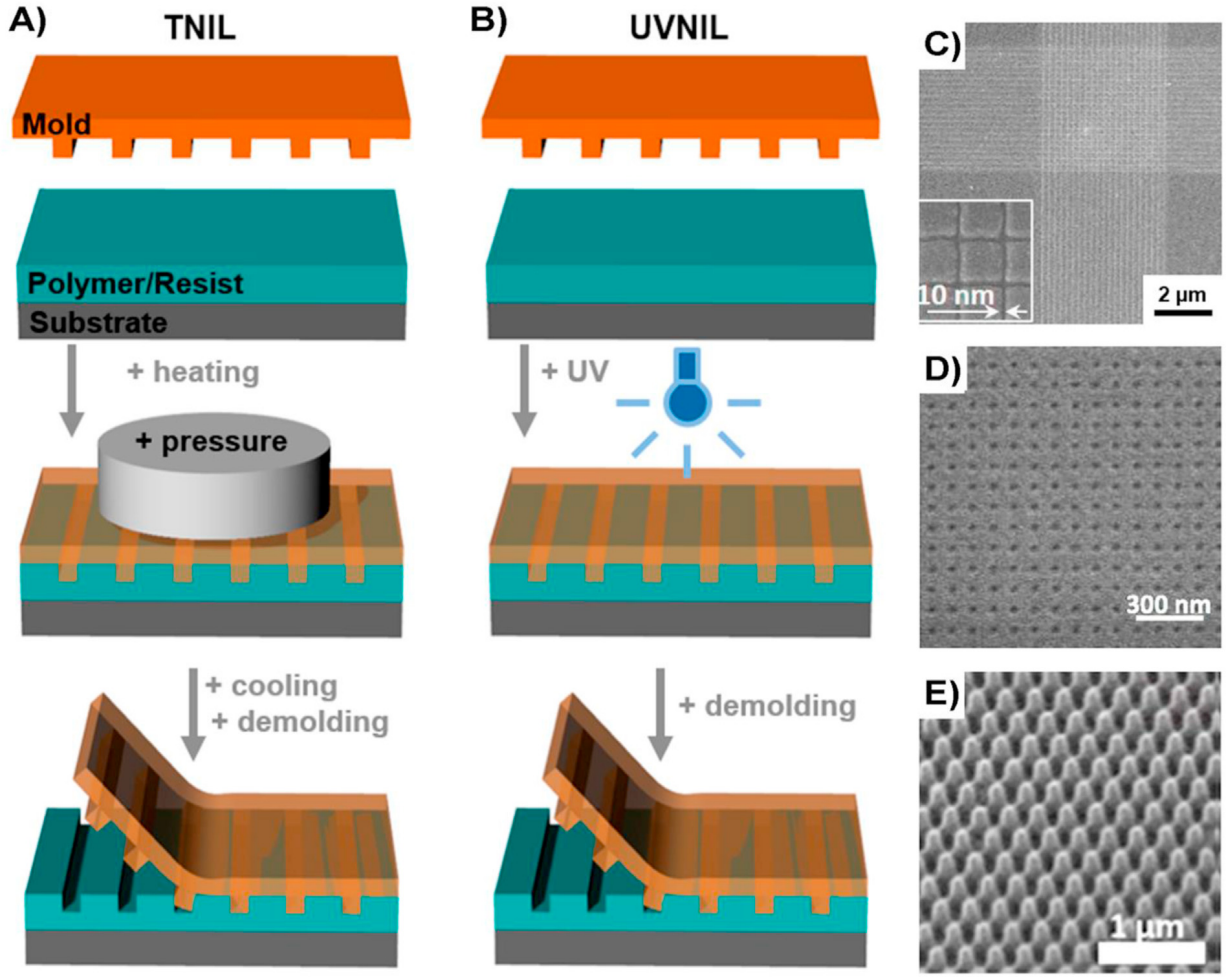
Schematic illustration of NIL process. A) Thermal heating (TNIL) uses heating and pressure for hardening and pattern transfer and B) Ultraviolet (UVNIL) uses UV light. C-E) Scanning electron microscopy images of nanotopographies of various shapes and sizes, that were successfully transferred to different substrates using NIL. Images modified from [60] under Creative Commons license.

The most common techniques for regular patterning creation with high level of reproducibility and regularity are lithographic techniques, where this term encompass different methods such as electron beam lithography [56], photolithography [57], dip-pen lithography [58], and nanoimprint lithography [59], each one of them having their advantages and disadvantages. For example, nanoimprint lithography (NIL) requires the use of a mould or template and a coated material or resist that can be easily moulded by a template (Fig. 2). The pattern transfer occurs during the resist hardening, most commonly using thermal heating (TNIL) or UV light (UVNIL) [60]. Lithographic techniques are normally combined with etching techniques such as reactive ion etching (RIE), so the pattern is completely transferred to the biomaterial substrate using the resist as a sacrificial protective mask [61]. RIE represents a dry etching technique, in contrast with wet etching, that avoids the need for liquids in contact with the surface, reducing the probabilities of residual contamination, which is paramount in biomedical applications [62]. A wide range of regular topographies can be fabricated using lithographic techniques, which allows in principle a more defined and reproducible control of the cell-surface interaction, compared with rough textured surfaces.

**Fig. 3:**
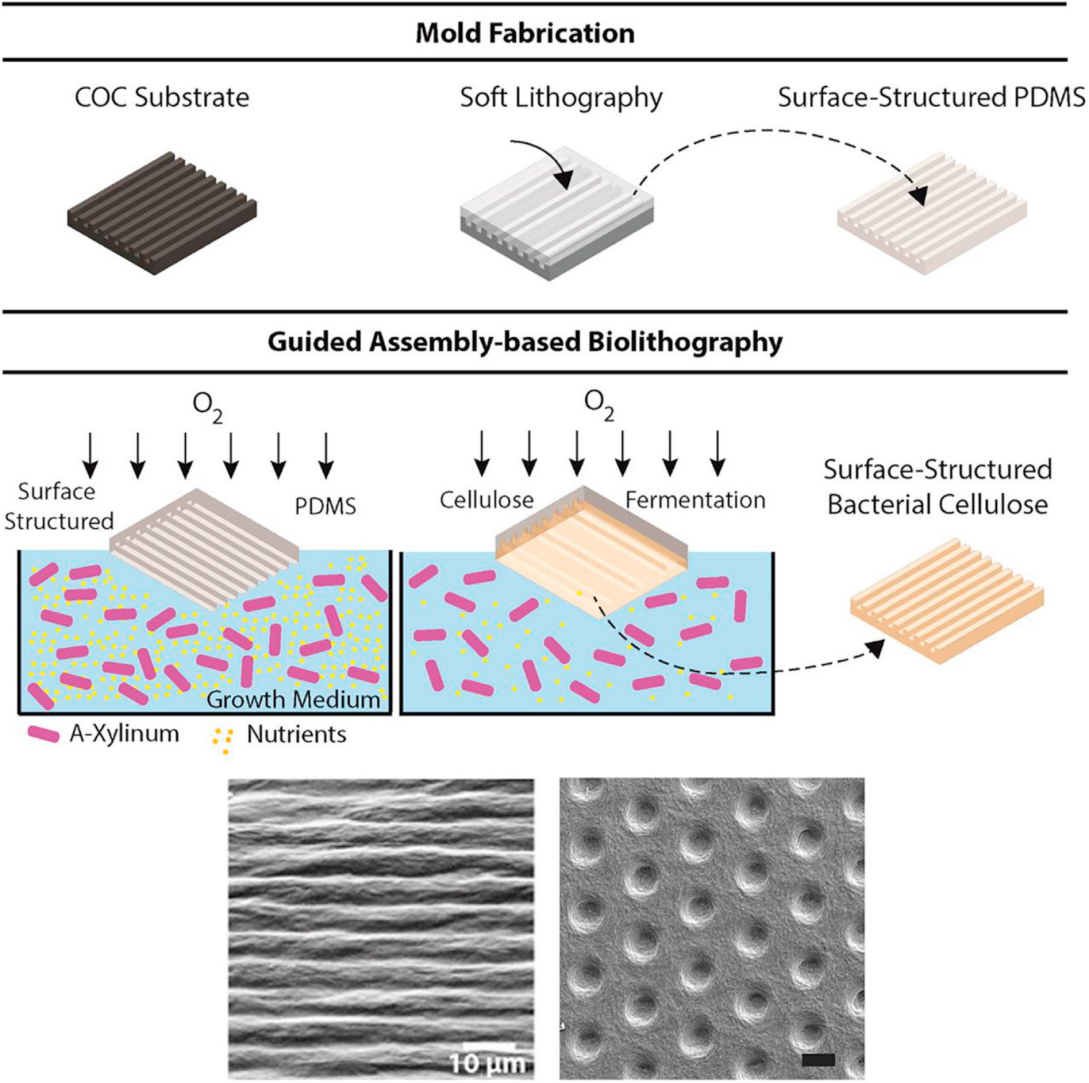
Schematic illustration of GAB nanofabrication process. Top: fabrication of PDMS mould using a template. Centre: the PDMS mould previously fabricated is used to cultivate cellulose as the bacterial growth media, thus allowing a patch of cellulose to develop on the top [64]. Copyright 2015, ACS. Bottom: Examples of nanopatterns that can be fabricated using GAB. Scale bars represent 10 ​μm [[64], [65]]. Copyright 2015, ACS. Copyright 2018, Springer Nature, under a Creative Commons Attribution 4.0 International.

Less conventional but promising lithographic techniques are emerging [63], aiming to reduce the cost of fabrication and ownership whilst maintaining pattern control and material versatility, i.e., guided assembly-based bio-lithography (GAB). GAB is a new technique that uses bacteria to cultivate cellulose on substrates, thus allowing fabrication of a ‘*surface-structured*’ cellulose patch (Fig. 3) [64].

Once the PDMS mould is peeled off, a negative replica is left on the surface of the cellulose. GAB is a very simple and cost-effective nanofabrication method which can be fully automated and achieve feature sizes as small as 10 ​nm. The use of bacterial cellulose is beneficial as this provides tuneable mechanical properties which can be modulated by changing the culture conditions. Bacterial cellulose patterned substrates fabricated by GAB were tested *in vivo* as skin dressing for skin regeneration, showing low inflammatory response over time [65].

### 3.2 Topographic roughness nanofabrication

Compared to regular/uniform topography, the introduction of roughness on implanted surfaces is easier to integrate on non-uniform surfaces and can be implemented for large-scale manufacturing at acceptable cost. Additionally, high level hierarchical structures can easily be achieved at both micro- and nano-scales, with the ability to replicate similar levels of hierarchy found in bone tissue; the aim being to improve bone tissue-implant compatibility [66]. However, rough surfaces can be difficult to control with a level of accuracy required for reproducible experiments, mainly due to the difficulty of controlling fundamental parameters (e.g. feature size, interspacing, alignment). Nevertheless, surface roughness can be quantified by measuring the degree of mean surface roughness (Ra) for a given surface [67], however, different topographies can be characterized by similar Ra values and yet can prompt different cellular responses to the surface. For this reason, there are many distinct parameters that can be used to define the ‘*surface roughness*’ with several nano-roughness techniques presenting opportunities for fabrication of a chosen topography or roughness value [68].

Gas-cluster ion beam (GCIB) is a versatile technique that uses pressurized argon gas due to its inert properties [69]. The gas is expanded through a small nozzle into vacuum to form a beam of gas clusters, where each cluster consists of a few hundreds to a few thousands of atoms or molecules of gaseous material that are accelerated by very high potentials (+10,000 ​V). With this technique, it is possible to create very fine features, including nanopores of a few atomic layers (≤10 ​nm) [70]. GCIB is compatible with polymers such as polyetheretherketone (PEEK) and common biocompatible metals such as titanium. An animal *in vivo* study using GAB as surface modification technique for PEEK disks, showed a 50% increase in bone growth increase covering treated PEEK, compared to no bone formation for the untreated control PEEK implants [71].

Selective laser sintering (SLS) technology uses laser beam for the surface treatment of biomaterials by a mechanism of ablation/vaporization. One of the main advantages is the formation of hierarchical textures at micro and nano levels without using potentially toxic materials in contact with the surface device, which could cause surface contamination and complications during the sterilization. SLS is compatible with a wide variety of commonly employed biomaterials, including Ti, its alloys, and biopolymers such as PEEK [72]. Schwartz et al., study reported the fabrication of micro-/nano-rough surfaces on Ti alloy (Ti–6Al–4V) and the *in vivo* study for enhanced vertical bone ingrowth [73]. Their findings revealed that hierarchical nanostructures promote vertical bone growth*,* and these implants could increase osseointegration in challenging patient cases.

**Fig. 4:**
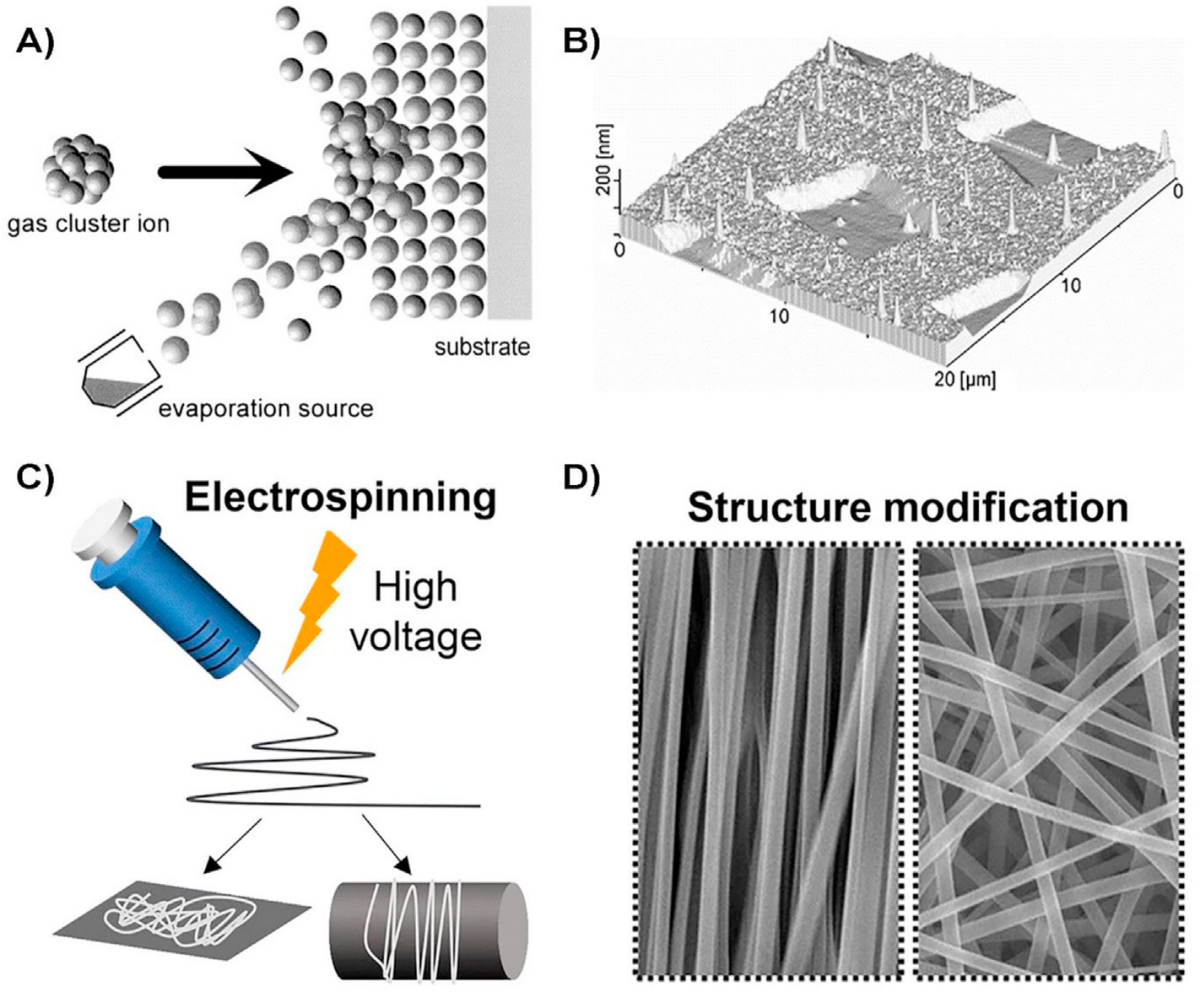
Schematic illustrations of GCIB and Electrospinning processes. A) GCIB process and B) example of the type of patterns that can be obtained on metal surfaces (Au) after GCIB process [70]. Copyright 2001, Elsevier. C) Nanofiber formation during the electrospinning process and D) scanning electron microscopy images of fibers with different orientation that can be obtained using electrospinning. Figure modified from [74] Copyright 2019, Springer One, under a Creative Commons Attribution 4.0 International.

Electrospinning applies an electric force during the material extrusion to fabricate continuous fibrous hierarchical nanostructures of different degree of complexity that can cover the surface of the biomaterial (Fig. 4c) [74]. High aspect ratio nanofibers with high alignment and diameters down to 200 ​nm can be easily fabricated for tissue engineering applications [75]. For example, aligned PCL/collagen nanofibers that biomimic the ECM of skin and thus promoting healing processes, can be fabricated by electrospinning [76]. Additionally, *in vivo* efficacy testing of electrospun PCL-polyexylthiophene nanofibers revealed that the aligned nanofibers polarised and increased the proliferation rates of fibroblast compared to the conventional (randomly orientated) nanofibers [77].

The plasma-spray technique involves the projection of precursor materials, heated by an electrical or arch plasma using argon or oxygen as gas feed at different pressure conditions [78]. It is widely used for the coating of metal implants such as Ti and Co–Cr alloys with hydroxyapatite (HA) to induce improved osseointegration [79,80]. Wet or chemical etching is a contact technique the requires abrasive agents as acids, e.g. H_2_SO_4_, HF or HCl or inorganic bases such as NaOH. These methods also alter surface wettability due to variation of surface chemistry [81], in synergy with topographic changes that can induce super-hydrophilic surface characteristics, but occasionally also non-wettable surfaces imposing great effects during the initial stages of cell-surface interaction. Overall, numerous studies have suggested an enhanced cellular response due to synergistic combination of topography and chemistry [82,83]. Chemical etching can be precisely controlled, to the level that several degrees of isotropic etching can be achieved with biopolymers, i.e., polyhydroxyvalerate (PHB), poly-l-lactic acid (PLLA) and metals, e.g. Mg [84] and it is alloys, Ti and its alloys [85]. Compared to dry etching, wet etching is a more cost effective and simplified process, although there is more risk of surface contamination. Sandblasting (or grit-blasting) is an abrasive surface modification technique that consist of applying high pressure to a stream of abrasive material against a surface. Commonly used abrasive materials are metal oxides such as Al_2_O_3_, TiO_2_, ZrO_2_ or crushed glass. Sandblasting is very versatile and is compatible with metals such as Ti and Ti alloys [86], and biopolymers such as PEEK and PLLA. This technique is usually combined with other techniques [87] as experimental evidence suggests an improved surface biocompatibility. For example, sandblasting and acid etching were combined to modify the topography and morphology of Ti alloy implanted into the femora of rabbits, showing an increased bone-contact implant after 4 weeks compared to control experiments [88].

## 4 High through-put screening (HTS) for topographical assessment and optimization

Due to our current limited understanding of the mechanism through which surface topography affects tissue behaviour, and small changes in nanotopographic parameters have effect on cell behaviour, high through-put platforms have allowed rapid screening of multiple topographies in parallel [89]. This section discusses recent studies that have developed or utilised a non-bias HTS approach to optimise nano-topographical precision for cell culturing research [90].

**Fig. 5:**
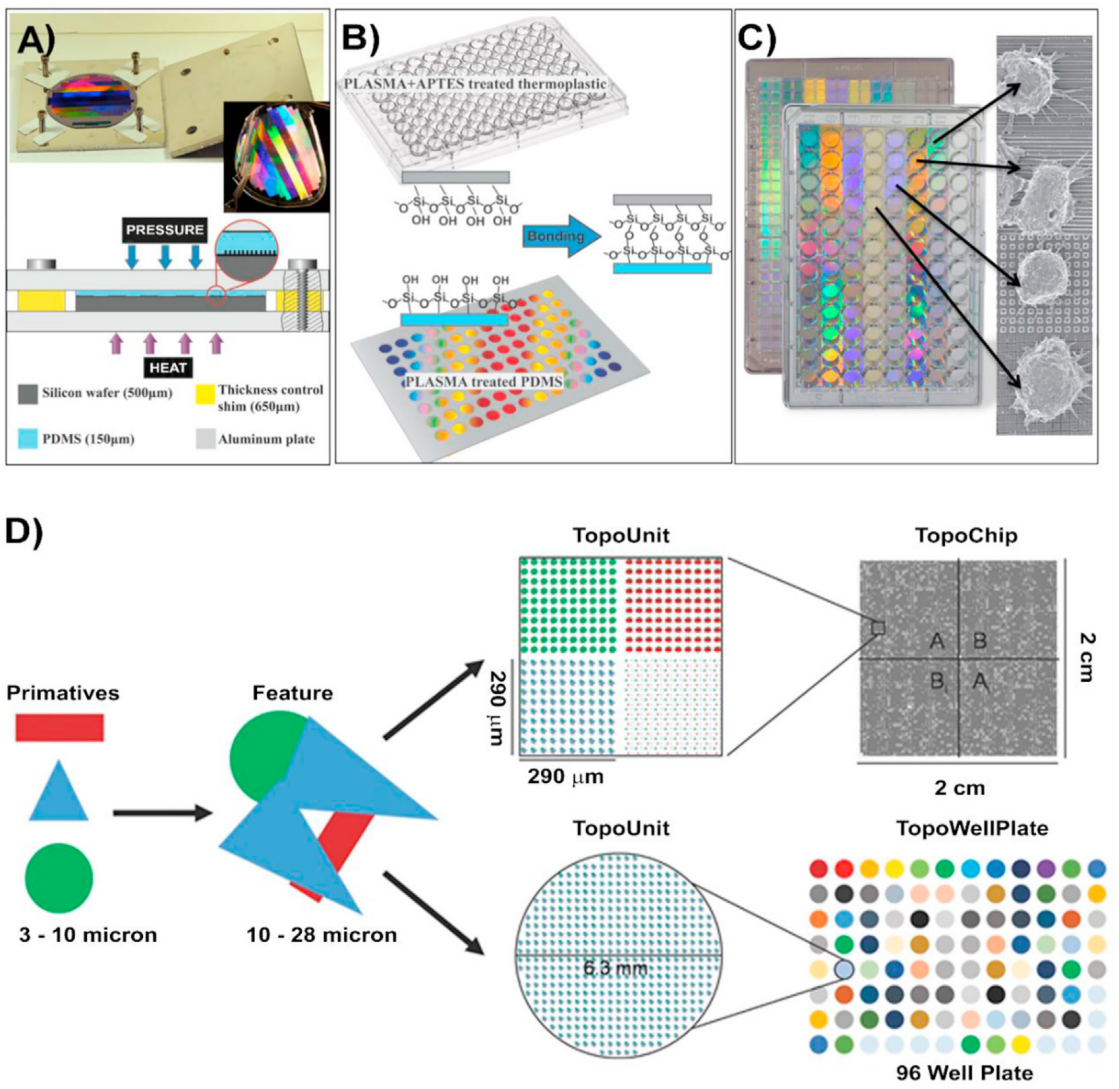
IMP and Nanotopochip platforms. (A) IMP fabrication process showing bonded plates assembly. The PDMS thickness is controlled by changing the relative thickness of Si wafer and the shims in four corners. (B) The patterned areas are then bonded to a multi-well plate thermoplastic plate previously treated with O_2_ plasma. The bottom surface of the plate is composed of PDMS nanotopographies. (C) Final integrated mechanobiology platform, in a 96 well plate configuration, enabling high through-put cell response analysis [92]. Copyright 2016, ACS. (D) TopoWellPlate is part of the TopoChip platform: Collectives of primitive shapes are used to design topographical features. Arrays of a unique topographical feature built in a 290 ​× ​290 ​μm TopoUnits, giving a total of 2176 TopoUnits with unique topographies and 4 unpatterned units for each Topochip. TopoWellPlate contains 87 unique surface topographies and 9 unpatterned wells incorporated by thermal bonding [93b]. Copyright 2017, John Wiley and Sons Inc.

Many studies have started to use topographical arrays as platforms for HTS to find novel correlations between topographical parameters and cell response, (see Fig. 5). These HTS platforms are advantageous for being cost-effective (financial and time), and significantly reducing the number of experiments required for high-quality non-bias results that can be compared more accurately [91]. Four HTS systems are available (Table 2): integrated mechanobiology platform (IMP) [92], NanoTopoChip [93] (Fig. 5), bioSurface structure array (BSSA) [94] and multi-architectural chip (MARC) [95].

**Table 2:** Comparison of the 4 different HTS systems developed as platforms for parallel screening topographies. Table adapted from [92].

	IMP	NanoTopoChip	BSSA	MARC
Pattern format	Single or multiple patterns per well. 96, 384 well plate.	2176 algorithm generated patterns on 2 ​cm ​× ​2 ​cm area. Primitive shapes: circles, triangles etc.	504 different patterns covering 3 ​mm ​× ​3 ​mm area.	6 ​× ​6 array of 18 patterns on 2.2 ​cm ​× ​2.2 ​cm
Transmission microscopy	YES	YES	NO	NO
High NA microscopy	YES	NO	NO	NO
Form factor	Tissue culture plate	Microarray chip	Microarray chip	Microarray chip
Automatic fluidic handling	YES	NO	NO	NO
Plate scanner read out	YES	NO	NO	NO

IMP is a very versatile platform that combine chemical factors and topography. The template used is fabricated by a silicon mould, previously created by electron-beam lithography and plasma etching. PDMS is then moulded and irreversibly bonded to standard bottomless microplates. The platform is compatible with multi-well plate-based instrumentation required for high through-put analysis. NanoTopoChip is a high parallel screening platform with a unique combination of 2176 different topographies on a single chip. NanoTopoChip master templates, fabricated by photolithography followed by dry etching, act as a template for chip replication using NIL. This platform was used to demonstrate the surface-cell response correlation *in vivo*, showing that some topographies induce a high expression of alkaline phosphatase in primary human mesenchymal stromal cells, which would promote bone bonding [96]. This study indicated that the combination of *in silico* design and HTS is a predictor for *in vivo* bone bonding, supporting previous similar findings. Although these results are promising, further research is needed to correlate *in silico* data and HTS approaches to *in vivo* studies, as very little is reported in this area [97,98]. BSSA is another platform that consists of a 3 ​× ​3 ​mm area with 504 different microstructures fabricated in PDMS by photolithography and dry etching [99]. BSSA has been used to evaluate topographies enabling proliferation or directed differentiation of undifferentiated embryonic stem cells (ES) [83]. Multi-architecture chip (MARC) allows the fabrication of PDMS chip microarrays of 2.2 ​cm ​× ​2.2 ​cm with 18 distinct surface topographies assembled onto a single surface, fabricated via NIL [84]. Various micro/nanopatterns of variable complexity can be imprinted, including isotropic and anisotropic features (i.e. pillars, holes, nano- and micrometer gratings) as single or hierarchical structures. This platform was used to study embryonic stem cells ​(hESCs), finding that some anisotropic topographies maximize the efficiency of neuronal differentiation from pluripotent stem cells [100].

## 5 Clinical and commercial stage

In this section we will overview the status of the most advanced studies on implanted devices with designed topography at pre-/commercial or clinical stage, for both soft and hard tissue applications (Table 4). There is a clear contrast between the number of patents published in the field and the number of clinical trials and marketed products, which highlights the inherent difficulty of translation from fundamental investigative *in vitro* experiments to *in vivo* clinical use. Table 3 highlights implant device clinical trials where the devices present nanotopographical modification of the surface. For clarity, in the case of this review surfaces presenting nanotopographical modification were taken as those with at least one dimension between 1 ​nm and 1 ​μm, with features in alignment with the definition of nanoscale outlined by regulatory bodies like the European Medicines Agency (EMA), Food and Drug Administration (FDA), and Health Canada [101].

**Table 3:** Current clinical trials, ongoing or recently completed, on implanted devices with nanotopography. Data source: clinicaltrials.gov.

Clinicaltrials.gov Identifier	Company	Technology	Fabrication method	Treatment
NCT00782171	Straumann AG	SLActive	SA	Dental implant
NCT04418830	NuVasive	Interbody implants	Laser sintering	Thoracic, Lumbar
NCT03649100	Hiossem	ETIII NH	SA	Dental implant
NCT03582657	Biotech dental	KONTACT N	SA	Dental implant
NCT04383834	NobelBiocare	TiUltra™	Anodization	Dental implant

### 5.1 Soft tissue applications

**Fig. 6:**
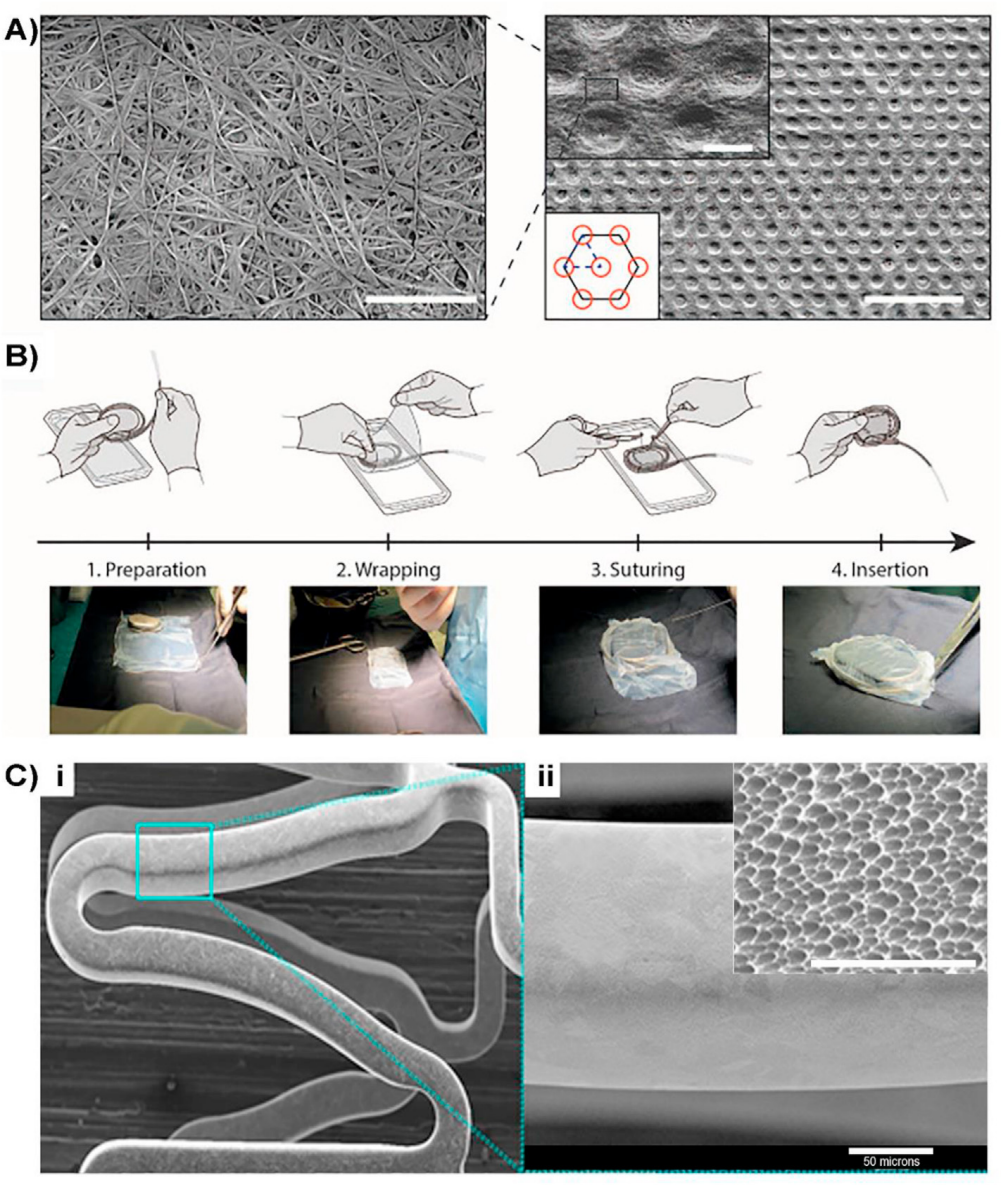
A) Scanning electron microscopy images of implanted micro-structured pouch fabricated using CellSense technology: Left: Image showing the fibrous structure (scale bar 2 ​μm) Right: Micro-well pattern on BC surface. Top-right inset detail of the micro-wells, where the fibrous structure of the material is evident. Bottom-left inset top-view schematic of the layout of the micro wells on the surface. Right inset: Red circles represent the micro-wells, solid black line depicts the hexagonal arrangement of the features and the blue dashed line highlights the elemental cell shaped as an equilateral triangle with 10 ​μm side length. Scale bar 300 ​μm. Scale bar in the inset 5 ​μm. B) Process chart of the surgical intervention during the *in vivo* implantation of the seamless pouch [102]. Copyright 2020, Science Direct. Ci) Scanning electron microscopy image of Nano+™ polymer-free stent nanotopography. Cii and inset) showing the size of the nano pores at a magnification of×20,000 and ​× ​6000 (inset). Figure modified from [105]. Copyright 2020, John Wiley and Sons Inc. (For interpretation of the references to colour in this figure legend, the reader is referred to the Web version of this article.)

Taming the inflammatory response with surface nanotopography during tissue regeneration can dramatically improve the functional outcomes of enhanced soft tissue biocompatibility and improved tissue regeneration. A reduced inflammatory response for implanted hernia repair mesh was demonstrated by surface modification developed by Exogenesis. NanoMesh™ hernia repair incorporates a nanotextured surface with feature sizes ranging from 20 to 50 ​nm, fabricated using an adaptation of the GCIB technique [62]. The polypropylene surface modified mesh exhibit enhanced soft tissue biocompatibility, tissue regeneration, reduced inflammatory response and improved anti-bacterial qualities. Despite of currently being in the pre-commercialization stage, the company is expecting to commercialise the implant later in 2021. Another product under development for the control of FBR, targeting towards the reduction of the fibrotic tissue around cardiac implantable electronic devices, is the CellSense technology developed by Hylomorph, (Fig. 6A and B). The implantable seamless pouch is fabricated using GAB for a fine control of the surface topography, consisting of a regular isotropic distribution of micro-sized wells with hexagon shape with a depth of 1.4 ​μm. The surface topography promotes a disruption in the adhesion and activation of immune cells through mechano-transduction. Long-term *in vivo* studies in animals have shown a significant reduction of fibrotic encapsulation when combined with a variety of cardiac medical devices [102]. The product is still under development and not yet commercialised.

Another class of cardiac implantable devices are the drug eluting stents (DES) for the treatment of patients with symptomatic coronary artery disease. The Nano+™ polymer -free stent (Lepu medical) consists of a stainless-steel platform and high-pressure delivery. In principle, the polymeric-free nature of Nano+™ prevents the adverse effects associated with the polymeric coating [103]. The stent surface can be prepared by several methods according to their patent [104], including chemical corrosion, electrochemical corrosion, anodic oxidation, micro-arc oxidation or micro-arc nitridation. The nanoporous cavities, with an average pore diameter of 400 ​nm, are distributed uniformly on the abluminal stent surface and functioning as a local drug carrier to improve early arterial healing, thereby reducing the risk of late stent thrombosis (Fig. 6C). A 1-year clinical study on the use of Nano+™ stent demonstrated similar safety and efficacy to commercialised polymeric stents [105].

Despite that most of the topographies used at research and clinical level are synthetic, natural topographies originally from the extracellular matrix are proven advantageous over their synthetic counterparts in cardiac applications. Cor^TM^PATCH epicardial patch commercialised by Cormatrix, is approved by the FDA for cardiac epicardial placement for support and repair of weakened areas of the heart [106]. The patch is extracted from regenerated native tissue, harvested from porcine small intestinal submucosa and subsequently decellularized (Table 4), resulting in a tissue with inherent micro and nanotopography, derived from the extracellular matrix, and used for cellular ingrowth with optimised angiogenesis and minimized inflammation [107].

**Table 4:** List of companies that have pre-/commercialised medical implants with nanofabricated surface, and their methods of fabrication.

Company	Technology	Fabrication method	Use
Exogenesis	nanoMesh™ 20–50 ​nm texture	Cluster Ion Beam (GCIB)	Hernia repair, enhanced biocompatibility
Hylomorph Medical	Cellsense Microsized wells (∼1.4 ​μm)	Guided assembly-based bio-lithography (GAB)	Low adhesion pouch for cardiac implantable electronic devices
Lepu Medical	Nano+™ ∼400 ​nm nanopores	Corrosion/Oxidation	Drug eluting stents Improved early arterial healing
Cormatrix	Cor^TM^PATCH Micro/nanotopography	Decellularization	Patch for Cardiac repair
Straumann Group	SLActive® Ra ∼1.5 ​μm	SA	Dental implants Accelerated osseointegration
Hiossen	ETIII NH Implant	SA	Dental implants Accelerated osseointegration
NuVasive	PEEK™ Pores	Laser sintering	Spinal implants Greater neck disability index and reduced pain
Aesculap	Plasmapore^XP^ Micro/nanoscale pores	Plasma Spraying	Spinal implants greater amount of tissue ingrowth
Nanovis	Nano FortiCore® 70 ​nm nanotubes	Chemical Vapour Deposition and anodization	Spinal implants enhanced bone regeneration
Tyber Medical	BioTy™	Plasma Spraying	Spinal implants reduced number of bacterial associated infections

### 5.2 Hard tissue applications

From a clinical perspective, the need for an increased implant success, especially in cases of low bone density or for other diseases, could be overcome with surfaces capable of promoting faster osseointegration [108]. Dental implants with designed roughness are known that have greater osseointegration rate compared with implants with smooth surface [109], with the upper limits for Ra can be considered below 2 ​μm [110]. For example, SLActive® (Straumann group) is a dental implant with Rã1.5 ​μm, and optimised nanotopography, fabricated by SA, that substantially promotes an accelerated osseointegration. A reduced healing time, from implant placement to implant loading, is achieved even in patients with challenging treatment protocols, such as diabetes and after radiation therapy treatments, where osseointegration is considerably less successful [111]. SA surface treatment can be combined with additional surface functionalisation strategies, modifying the surface properties of the surface. ETIII NH dental Ti implants, commercialised by Hiossen, contain a nano-layer of bioresorbable apatite that modifies the physical and chemical surface properties, rendering a superhydrophilic surface which improves osseointegration in the early healing stages by providing more blood contact and boosting healing times. Clinical studies have shown an increased implant stability measured as implant stability quotient [112]. More efforts in the dental field at clinical level are ongoing, with several clinical trials identified (Table 3). Clinical trial number NCT04383834 for example, is focused on studying the effect of gradual change in topography, in combination with surface chemistry, on early osseointegration and designed bone stability of superhydrophilic dental implants.

**Fig. 7:**
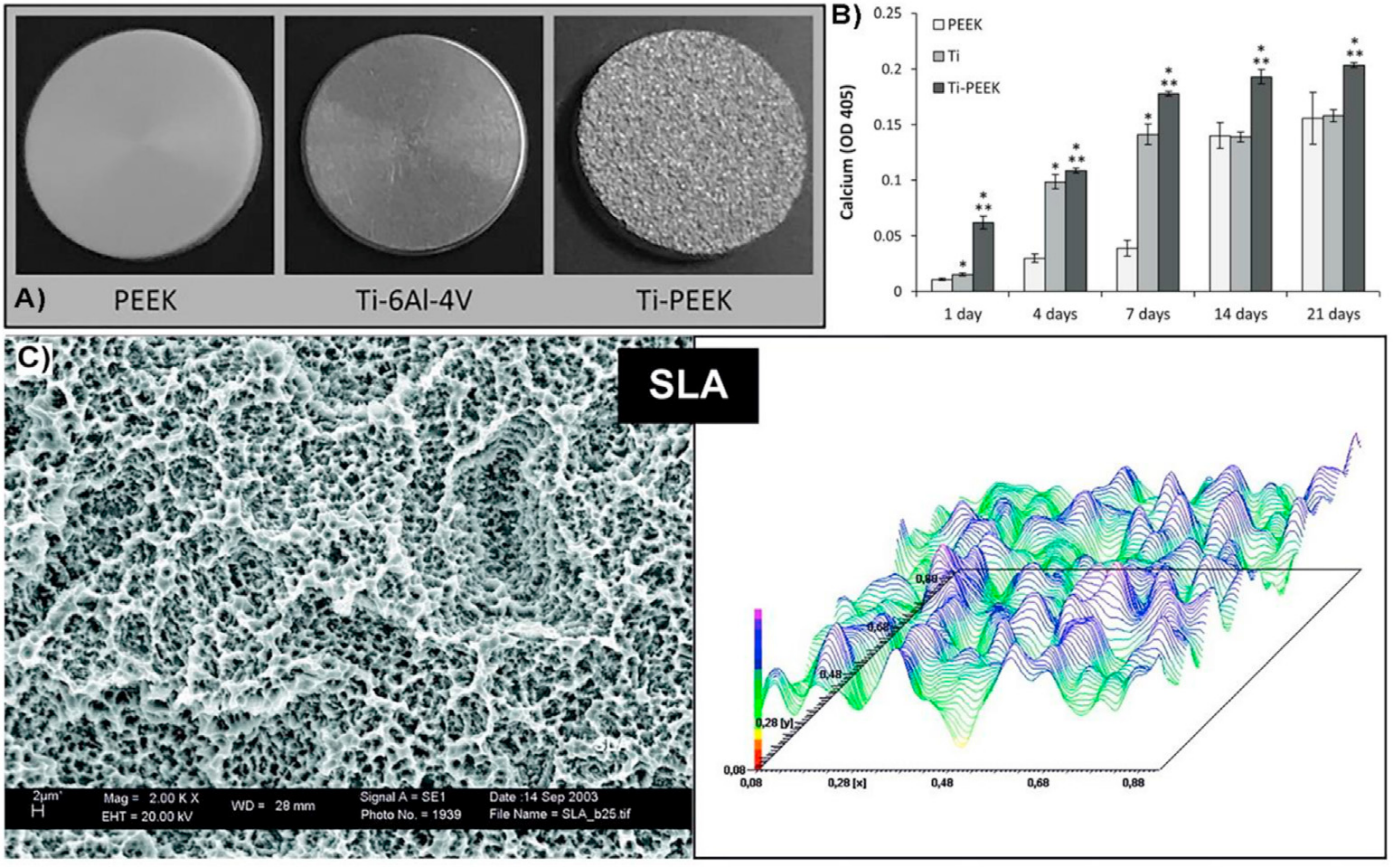
Ti-PEEK spinal implants with hierarchical roughness. A) Pictures of implanted samples used to study the influence of hierarchical roughness with calcium deposition compared to other surfaces and B) Graph showing *in vitro* variations in the calcium deposition comparing the 3 implanted surfaces, showing the Ti-PEEK implant (TyPEEK, Tyber Medical) with the highest increased calcium deposition in presence of human osteoprogenitor cells [117]. Copyright 2019, Science Direct, under a Creative Commons Attribution 4.0 International. C) Right: SEM image of micro and nanoscale topography achieved using SLA on coated titanium plasma-sprayed PEEK for spinal implants. The hierarchical structure is composed by cavities with diameters of wider indentations of about 10–50 ​μm completely superposed by smaller pores of about 1–2 ​μm diameter. Left: profilometric contact style topography representing the surface waviness. Figure modified from [116]. Copyright 2005, John Wiley and Sons Inc.

**Fig. 8:**
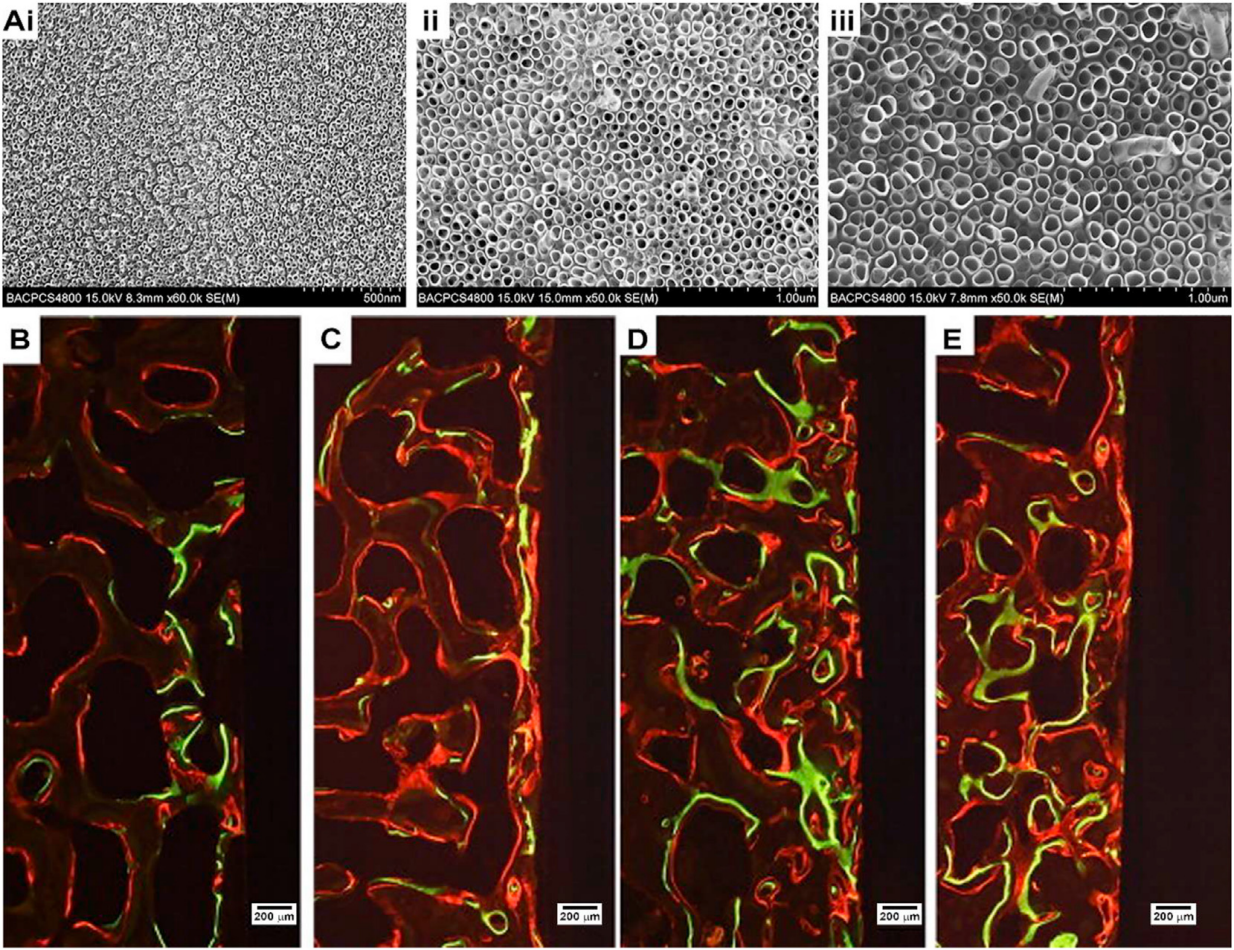
Ti-Coated PEEK spinal implants. A) SEM images of spinal implant (nano FortiCore®, Nanovis) coated with TiO_2_ nanotubes with different pore diameters: i) 30 ​nm, ii) 70 ​nm, iii) 100 ​nm. B, C, D, E) Fluorochrome images of surrounding tissue of spinal implants showing new bone formation around all implants modified by (B) machined implant, (C) coated with 30 ​nm TiO_2_ nanotubes, (D) 70 ​nm TiO_2_ nanotubes and (e) 100 ​nm TiO_2_ nanotubes, with different markers: xylene orange-labeled lines (orange), calcein-labeled lines (green), alizarin-labeled lines (red). Figure modified from [118]. Copyright 2011, ScienceDirect. (For interpretation of the references to colour in this figure legend, the reader is referred to the Web version of this article.)

Increased osseointegration is fundamental in spinal applications for an effective implant integration, and PEEK and Ti can be considered the standard materials. PEEK is a high-performance polymer with excellent mechanical properties for spinal applications and better radiographic visualisation compared to metals. Since smooth PEEK has limited implant osseointegration, the incorporation of nanopores promotes a bone in-growth [113]. Several commercialised products have incorporated nanotopography for better osseointegration; for example, laser sintering in combination with 3D printing technology is employed to fabricate nanopores onto PEEK™ spinal interbody constructs (Nuvasive) [114]. Clinical studies for PEEK™ have shown a significantly greater neck disability index and neck pain improvement with porous PEEK devices when compared to patients receiving smooth PEEK devices and sustained through 12 months post-op. Plasmapore^XP^ (Aescular implants) also incorporates porosity at microscale, in combination with nanoscale features, for an improved osseointegration and implant anchorage. The Ti-PEEK implant surface is the result of a two-stage coating process applied to the surface of the PEEK substrate. The combination of the surface activation of the PEEK material and the vacuum plasma spray (VPS) coating on the sandwiched pure titanium layer, generates an adhesive composition on the PEEK substrate. The results of the histological portion of the *in vivo* study showed that the Ti-PEEK implants demonstrate a significantly greater amount of bone ingrowth at 12 weeks when compared to the uncoated PEEK implants [115]. Hierarchical roughness at micro and nanoscale was also achieved on coated titanium plasma-sprayed PEEK for spinal implants (TyPEEK®, Tyber Medical) [116]. Consequently, the formation of hierarchical roughness increases the surface area which results in an increased calcium deposition compared to smoother surfaces (Fig. 7) [117]. Similar strategy of increasing surface area for enhanced bone regeneration, is used for Nanovis in its commercialised spinal implants through their Ti-PEEK nano FortiCore® interbody line, coated with TiO_2_ nanotubes (Fig. 8). The implants are composed of a highly porous titanium scaffold integrated with the core implant by anodization. Pre-clinical studies on porcine model showed favourable molecular response and enhanced osseointegration modulated by the diameter of the TiO_2_ nanotubes [118].

Bacterial infections are a common cause of implant failure, involving a huge cost associate with their removal [4a]. Surface treatment in different types of biomaterials [119,120], including metallic implants, can generate nanotextures and reduce the number of bacterial associated infections [121] and this strategy has reached the clinical stage. For example, BioTy™ implants (Tyber Medical) consist of surface modified Ti spinal implants by plasma spraying to produce nanotextures with antibacterial properties. The use of designed topography alone for the control of bacteria is proven to result in a statistically significant reduction of common pathogens such as Staphylococcus Aureus, Pseudomonas Aeruginosa and E. Coli. These results confirm the numerous *in vitro* studies showing that nanotopography alone is an effective method to control bacteria colonization [122]. Although this new technology is promising, as circumvents the need of chemical cues, is yet to be commercialised.

## 6 Summary and outlook

The perpetual need for the development of safer and more sophisticated devices has propelled a remarkable control of the immune response to foreign materials that has guided in the design of implants with improved tissue integration. Yet, a more in-dept analysis of cell tissue-surface interactions is still required, since most of the advances accomplished are based on a heuristic approach. Despite of the current challenging factors facing the scientific community (e.g. the lack of a clear picture of the underlying mechanism, engineering challenges, inherent difficulty of translation from *in vitro* to *in vivo*), successful examples of implants can be found in diverse biomedical fields, and this family will probably be expanded in the future. Conversely, the number of clinical fields with surface engineered implants at clinical stage can be considered still scarce, if we compare it to the untapped potential of the surface modifications of biomaterials shown at *in vitro* level. Only a few of the current patterning techniques available have been adapted at commercial scale, and in most of the cases, are employed for surface roughness modification, thus limiting the options available for regular topographic patterning. Therefore, novel nanopatterning technologies will be required, if we want to open new avenues, orientated to a better translation between research and clinical use; we aim to introduce more complex and regular patterns, since most of the topographies tested in clinical trials are simple [123]. And while hard tissue applications such as dental and spinal implants are dominating the new products, applications for soft tissues are starting to emerge, incorporating more refined topographies and new technologies, which could further open up new and exciting avenues to explore in further medical fields. The economics, cost-effectiveness, scale-up potential, and robustness of the patterning techniques are also fundamental from a commercialization point of view. Future avenues with plenty of room for innovation in implants are several: 1-the development of the next generation of nanopatterning techniques that could pave the way to overcome one of the bottlenecks of transferring very defined patterns with controlled defectivity to whole 3D medical implants or only specific areas. 2-machine learning could shed light on the unknown complex relationships with the environment that translates into genetic modifications [124]. 3-the synergetic combination of techniques for the design of surfaces with chemical/biochemical cues and topography, where these topographies could be hierarchically designed, i.e. micro- and nanotopography, 2D and 3D nanopatterning [125]. 4D designed scaffolds for tissue engineering [126] and regenerative medicine represent a very promising field with a yet to define potential that will become more relevant in the future, and is expected to reach commercial applications in this decade [127]. Beyond the inherent challenges outlined, new regulatory environments such as the newly introduced European medical regulation is expected to add additional burden to the introduction of new implanted devices at the commercial stage [5]. Nevertheless, we can conclude that this area is still gaining significant traction and we anticipate an expansion towards more clinical fields of application in the future.

## Declaration of competing interest

The authors declare that they have no known competing financial interests or personal relationships that could have appeared to influence the work reported in this paper.
